# Patients with early rheumatoid arthritis exhibit elevated autoantibody titers against mildly oxidized low-density lipoprotein and exhibit decreased activity of the lipoprotein-associated phospholipase A_2_

**DOI:** 10.1186/ar2129

**Published:** 2007-02-27

**Authors:** Evangelia S Lourida, Athanasios N Georgiadis, Eleni C Papavasiliou, Athanasios I Papathanasiou, Alexandros A Drosos, Alexandros D Tselepis

**Affiliations:** 1 Department of Chemistry, Laboratory of Biochemistry, University of Ioannina, 45110 Ioannina, Greece; 2 Department of Internal Medicine, Medical School, University of Ioannina, 45110 Ioannina, Greece

## Abstract

Rheumatoid arthritis is a chronic inflammatory disease, associated with an excess of cardiovascular morbidity and mortality due to accelerated atherosclerosis. Oxidized low-density lipoprotein (oxLDL), the antibodies against oxLDL and the lipoprotein-associated phospholipase A_2 _(Lp-PLA_2_) may play important roles in inflammation and atherosclerosis. We investigated the plasma levels of oxLDL and Lp-PLA_2 _activity as well as the autoantibody titers against mildly oxLDL in patients with early rheumatoid arthritis (ERA). The long-term effects of immunointervention on these parameters in patients with active disease were also determined. Fifty-eight ERA patients who met the American College of Rheumatology criteria were included in the study. Patients were treated with methotrexate and prednisone. Sixty-three apparently healthy volunteers also participated in the study and served as controls. Three different types of mildly oxLDL were prepared at the end of the lag, propagation and decomposition phases of oxidation. The serum autoantibody titers of the IgG type against all types of oxLDL were determined by an ELISA method. The plasma levels of oxLDL and the Lp-PLA_2 _activity were determined by an ELISA method and by the trichloroacetic acid precipitation procedure, respectively. At baseline, ERA patients exhibited elevated autoantibody titers against all types of mildly oxLDL as well as low activity of the total plasma Lp-PLA_2 _and the Lp-PLA_2 _associated with the high-density lipoprotein, compared with controls. Multivariate regression analysis showed that the elevated autoantibody titers towards oxLDL at the end of the decomposition phase of oxidation and the low plasma Lp-PLA_2 _activity are independently associated with ERA. After immunointervention autoantibody titers against all types of oxLDL were decreased in parallel to the increase in high-density lipoprotein-cholesterol and high-density lipoprotein-Lp-PLA_2 _activity. We conclude that elevated autoantibody titers against oxLDL at the end of the decomposition phase of oxidation and low plasma Lp-PLA_2 _activity are feature characteristics of patients with ERA, suggesting an important role of these parameters in the pathophysiology of ERA as well as in the accelerated atherosclerosis observed in these patients.

## Introduction

Rheumatoid arthritis is a chronic inflammatory condition of unknown etiology affecting primarily the synovium, leading to joint damage and bone destruction [1]. Rheumatoid arthritis causes significant morbidity as a result of synovial inflammation, joint destruction and associated disability. Several investigators have reported an excess of cardiovascular morbidity and mortality among rheumatoid arthritis patients. In active rheumatoid arthritis, the majority of cardiovascular deaths result from accelerated atherosclerosis [2-5].

Oxidative modification of low-density lipoprotein (LDL) is an important event in the development and progression of atherosclerosis. Oxidized low-density lipoprotein (oxLDL) is present in atherosclerotic lesions of humans and animal models, and promotes atherosclerosis by several mechanisms [6-9]. oxLDL has been detected in patients with systemic lupus erythematosus and the antiphospholipid syndrome and also in the synovium and synovial fluids of rheumatoid arthritis patients [10,11].

During LDL oxidation both the lipids and apolipoprotein B-100 (Apo B) undergo a variety of chemical changes via radical-mediated reactions as well as modifications by chemically active products formed on oxLDL particles [12]. An important biochemical change that takes place during LDL oxidation is the hydrolysis of its content in oxidized phospholipids and the production of lysophosphatidylcholine. This reaction is catalyzed by the lipoprotein-associated phospholipase A_2 _(Lp-PLA_2_), also known as platelet-activating factor acetylhydrolase [13]. Lp-PLA_2 _exhibits a Ca^2+^-independent phospholipase A_2 _activity and preferentially hydrolyses biologically active phospholipids containing short acyl groups at the *sn-2 *position, such as platelet-activating factor and oxidized phospholipids [13]; this enzyme therefore plays important roles in inflammatory reactions and atherosclerosis [14]. In human plasma Lp-PLA_2 _is associated mainly with LDL, whereas a small proportion of circulating enzyme activity is also associated with high-density lipoprotein (HDL) [13,15]. Data from large Caucasian population studies have demonstrated an independent association between plasma Lp-PLA_2 _(which represents mainly the LDL-associated Lp-PLA_2_) and the risk of future cardiovascular events [16,17]. In contrast to the total plasma enzyme, several lines of evidence suggest that HDL-associated Lp-PLA_2 _activity (HDL-Lp-PLA_2_), although at low levels in plasma, may contribute to the antiatherogenic effects of this lipoprotein [13].

oxLDL is immunogenic and some of its constituents (oxidized phospholipids, aldehydes and lysophosphatidylcholine) play important roles in the oxLDL antigenicity, participating in the formation of several different epitopes. These epitopes are recognized by specific autoantibodies, which are present in serum of healthy individuals as well as in various pathologic conditions [18]. We recently showed, using various types of mildly oxLDL as antigens, that the extent of LDL oxidation and the levels of LDL-associated Lp-PLA_2 _activity significantly influence the antibody titers against oxLDL in patients with stable angina [19,20]. Furthermore, we recently showed that the LDL-associated Lp-PLA_2 _plays an important role in modulating the immune responses against various types of mildly oxLDL observed after an acute coronary syndrome without persistent elevation of the ST segment [21].

The aim of the present study was to investigate the plasma levels of oxLDL and Lp-PLA_2 _activity as well as the autoantibody titers against various types of mildly oxidized LDL in patients with early rheumatoid arthritis (ERA). The long-term effects of immunointervention on these parameters in patients with active disease were also determined.

## Materials and methods

### Patients

Fifty-eight consecutive patients with ERA (14 men and 44 women) who met the American College of Rheumatology 1987 criteria for rheumatoid arthritis [22] and 63 apparently healthy nonsmoking volunteers (controls) were investigated. ERA patients were >18 years of age and had early inflammatory disease (disease duration <12 months) without prior use of disease-modifying antirheumatic drugs (DMARDs) and/or corticosteroids. All patients were recruited from the outpatient rheumatology clinic of the University Hospital of Ioannina, Greece. Details on the eligibility criteria for inclusion or exclusion from the study were reported in our previously published prospective, controlled study [23].

ERA patients were treated with methotrexate (0.2 mg/kg/week), and prednisone (7.5 mg/day). The dose of methotrexate remained stable during the study, while the dose of prednisone was tapered to 5 mg/day according to the patients' clinical response. Disease activity was assessed by measuring the disease activity score for 28 joint indices [24], while the clinical response was evaluated according to the American College of Rheumatology 50% response criteria [25]. All patients were followed up every month for the first 3 months, and every 3 months thereafter. During the follow-up period, a questionnaire concerning changes in dietary habits was carefully completed by all patients. The body weight was also measured appropriately in each visit. Overnight fasting blood samples were obtained at baseline and after 12 months follow-up from both the ERA patients and the control group. The Ethics Committee of the University Hospital of Ioannina approved the study and written informed consent was obtained from each patient and each healthy volunteer.

### Measurement of autoantibody titers against oxidized low-density lipoprotein

LDL (density = 1.019–1.063 g/ml) was isolated by sequential ultracentrifugation from pooled fresh plasma [26]. LDL, at a final concentration of 100 μg protein/ml, was oxidized in the presence of 5 μM CuSO_4 _for up to 3 hours at 37°C under continuous monitoring of the increase in the absorbance at 234 nm, as we recently described [19,20]. Oxidation of LDL was terminated by the addition of 0.01% ethylenediamine tetraacetic acid either at the end of the lag phase (oxLDL_L_), at the end of the propagation phase (oxLDL_P_), or during the decomposition phase (oxLDL_D_), 3 hours after the onset of oxidation [19,20]. The serum autoantibody titers of the IgG type against all types of oxLDL were determined by an ELISA method, as we recently described [19,20]. The results were expressed as the ratio of antibody binding to various types of oxLDL versus LDL [19,20].

### Determination of lipoprotein-associated phospholipase A_2 _activity

The Lp-PLA_2 _activity in plasma and in HDL-rich plasma, after the sedimentation of all Apo B-containing lipoproteins with dextran sulfate–magnesium chloride (HDL-Lp-PLA_2_), was measured by the trichloroacetic acid precipitation procedure, using 1-*O-*hexadecyl-2- [^3^H-acetyl]-*sn*-glycero-3-phosphocholine as a substrate at a final concentration of 100 μmol/l. The reaction was performed for 10 min at 37°C and the Lp-PLA_2 _activity was expressed as nanomoles of 1-*O*-hexadecyl-2-acetyl-*sn*-glycero-3-phosphocholine degraded per minute per milliliter of plasma [20,27,28]. The minimum detection limit of the assay is 0.8 nmol/min/ml plasma, whereas the intra-assay and inter-assay coefficients of variation are 3.3–4.2% and 7.1–8.0%, respectively.

### Analytical methods

Serum lipids were determined after 12 hours overnight fast. Total cholesterol, triglycerides and HDL-cholesterol levels were determined on the Olympus AU560 Clinical Chemistry analyzer (Hamburg, Germany) as previously described [28]. LDL-cholesterol was estimated by calculation, using the Friedewald formula [29]. Serum Apo B and apolipoprotein A-I (Apo A-I) levels were measured by immunonephelometry with the aid of a Behring Nephelometer BN100 and reagents (antibodies and calibrators) from Behring Diagnostics GmbH (Liederbach, Germany). Serum lipoprotein (a) (Lp(a)) levels were determined by an enzyme immunoassay method (Macra Lp(a); Terumo Medical Corporation Diagnostic Division, Elkton, MD, USA) [27]. C-reactive protein (CRP) and IgM rheumatoid factor were measured by nephelometry. The erythrocyte sedimentation rate was measured by the modified Westergren method.

### Statistical analysis

Data were expressed as the mean ± standard deviation. Statistical analysis was performed using the paired Student's *t *test for comparisons between baseline and post-treatment values, while one-way analysis of variance followed by the least significant difference *post hoc *test was used for comparisons between individual groups. Comparison between baseline and post-treatment CRP levels was performed using the Kruskal–Wallis test while CRP levels between individual groups were compared using the Mann–Whitney U test. Correlation between variables was examined using the Pearson's correlation coefficient. We used multivariate logistic regression analysis to calculate the adjusted odd ratios and 95% confidence intervals for the two study groups.

Matched groups were constructed taking into account the significant factors as derived from the univariate logistic regression analysis. The model selection used the backward stepwise method (likelihood ratios), and variables at a *P *value less than 0.05 were retained in the model as independent variables. The variables included in the univariate analysis were the following: age, total cholesterol, LDL-cholesterol, HDL-cholesterol, triglycerides, Apo B, Apo A-I, autoantibody titers against oxLDL_L_, oxLDL_P _and oxLDL_D_, plasma Lp-PLA_2 _activity, HDL-Lp-PLA_2 _activity (continuous variables) and female gender (dichotomous variable). All statistical analyses were carried out with SPSS 12.0 (SPSS Inc., Chicago, IL, USA). In all cases, *P *< 0.05 was considered statistically significant.

## Results

### Patients' characteristics and lipid profile

Fifty-eight patients with ERA and 63 apparently healthy volunteers participated in the study. The clinical and biochemical characteristics of the study population are presented in Table 1. There was no observed difference in sex distribution, age and body mass index between ERA patients and controls. As expected, ERA patients exhibited increased levels of the inflammatory markers CRP and erythrocyte sedimentation rate and had a high disease activity score as measured by the disease activity score for 28 joint indices (Table 1). In addition, ERA patients exhibited a mild dyslipidemia characterized by an increase in the serum levels of total cholesterol, LDL-cholesterol, triglycerides and Apo B as well as by a decrease in the serum levels of HDL-cholesterol and Apo A-I compared with controls. No difference in the serum Lp(a) levels was observed between the two study groups.

One year of therapy with DMARDs in ERA patients resulted in a significant decrease of the inflammatory markers CRP and the erythrocyte sedimentation rate as well as in the reduction of the disease activity score for 28 joint indices (Table 1). In addition, one year of therapy with DMARDs resulted in a significant increase in the serum levels of total cholesterol, HDL-cholesterol and Apo A-I compared with the respective baseline values (Table 1). It should be noted that no female patient was receiving hormone replacement therapy either at baseline or during the follow-up period.

### Lipoprotein-associated phospholipase A_2 _activity

At baseline, ERA patients exhibited a significantly lower activity of total plasma Lp-PLA_2 _and of HDL-Lp-PLA_2_, compared with controls (Table 1). One year of therapy with DMARDs did not influence the total plasma Lp-PLA_2 _but it significantly increased the HDL-Lp-PLA_2 _activity (Table 1).

### Autoantibody titers against oxidized low-density lipoprotein

**Figure 1:**
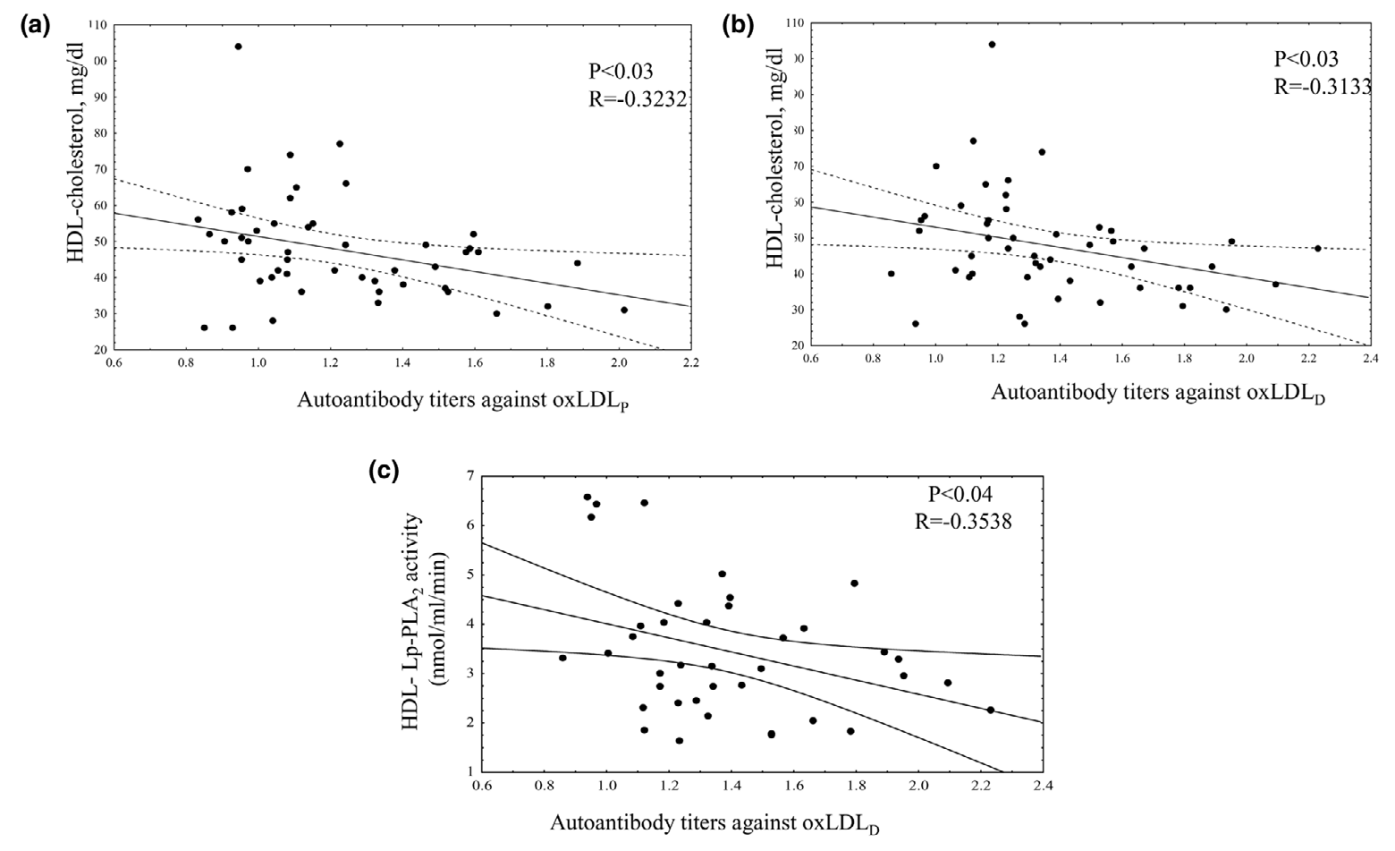
Correlation between serum levels of high-density lipoprotein-cholesterol and autoantibody titers against oxidized low-density lipoprotein. Correlation between serum levels of high-density lipoprotein (HDL)-cholesterol and autoantibody titers against **(a) **oxidized low-density lipoprotein in the propagation phase (oxLDL_P_) and **(b) **oxidized low-density lipoprotein in the decomposition phase (oxLDL_D_) in early rheumatoid arthritis patients at baseline. **(c) **Correlation between HDL-associated lipoprotein-associated phospholipase A_2 _(HDL-Lp-PLA_2_) activity and autoantibody titers against oxLDL_D _in early rheumatoid arthritis patients at baseline.

Three types of mildly oxLDL were prepared and used as antigens: oxLDL_L _at the end of the lag phase, oxLDL_P _at the end of the propagation phase and oxLDL_D _at the decomposition phase, 3 hours after the onset of oxidation. As shown in Table 2, ERA patients exhibited higher autoantibody titers against all types of oxLDL at baseline compared with controls. Importantly, the autoantibody titers against oxLDL_P _and oxLDL_D _were inversely correlated with serum HDL-cholesterol levels (Figure 1). In addition, autoantibody titers against oxLDL_D _were inversely correlated with HDL-Lp-PLA_2 _activity (Figure 1). One year of therapy with DMARDs resulted in a significant decrease in autoantibody titers against all types of oxLDL in ERA patients compared with the respective baseline values (Table 2).

### Association of autoantibody titers against oxidized LDL and plasma lipoprotein-associated phospholipase A_2 _with early rheumatoid arthritis

We initially performed univariate analysis using the lipid parameters that were significant different between ERA patients and controls, the antibody titers against the various types of oxLDL and the Lp-PLA_2 _activity, in order to evaluate their relationships with the presence of ERA. The results of this analysis showed that only autoantibody titers against all types of oxLDL as well as the low plasma Lp-PLA_2 _activity are associated with ERA (Table 3). To further identify whether these parameters could independently be associated with ERA, multivariate logistic regression analysis was performed, taking into account all statistically significant factors as they derived from univariate analysis. In the multivariate analysis model we therefore included the autoantibody titers against oxLDL_L_, oxLDL_P _and oxLDL_D_, and the plasma Lp-PLA_2 _activity as defined from univariate analysis. In this analysis ERA showed significant associations only with autoantibody titers against oxLDL_D _and plasma Lp-PLA_2 _activity (Table 4).

## Discussion

The present study shows for the first time that ERA patients exhibit low plasma Lp-PLA_2 _activity and elevated autoantibody titers against mildly oxidized types of LDL (oxLDL_L_, oxLDL_P _and oxLDL_D_), compared with controls. The low Lp-PLA_2 _activity is in accordance with previously published data by our group, indicating that patients with active juvenile rheumatoid arthritis presented with lower plasma Lp-PLA_2 _activity compared with those with inactive disease or to controls [30]. The present study further shows that the low Lp-PLA_2 _activity is independently associated with ERA. It is well established that the main cellular source of the plasma form of Lp-PLA_2 _is monocytes, which secrete this enzyme during their differentiation into macrophages [31]. The cellular expression of plasma Lp-PLA_2 _is regulated by various factors, including the differentiation state of the cell and the degree of activation by proinflammatory mediators [13,32]. Most of the proinflammatory mediators (lipopolysaccharide, tumor necrosis factor alpha, IL-1, IL-8, and interferon gamma) inhibit Lp-PLA_2 _expression by macrophages *in vitro *[13]. The reduction in plasma Lp-PLA_2 _activity found in ERA patients could therefore be attributed to the inflammation-induced decrease in the enzyme expression. According to our previously published results, however, another important determinant of the plasma Lp-PLA_2 _activity is the plasma LDL level [27,28]. Indeed, Lp-PLA_2 _in plasma is mainly bound on LDL particles, whereas a small proportion is associated with HDL [13]. We may consequently suggest that the low levels of enzyme activity in the plasma of ERA patients at baseline could be the combined effect of the inflammation-induced reduction of enzyme secretion from macrophages and the expected increase in plasma enzyme levels due to the elevation of LDL-cholesterol in plasma of ERA patients.

The dependence of the plasma Lp-PLA_2 _levels from the LDL-cholesterol levels could also explain our results showing that therapy with DMARDs did not affect either the plasma LDL-cholesterol levels or the plasma Lp-PLA_2 _activity. A factor that could also influence the plasma Lp-PLA_2 _levels in ERA patients is Lp(a). Indeed, we [33] and others [34] have previously shown that Lp(a) contains several-fold greater Lp-PLA_2 _activity compared with LDL when assayed at equimolar protein concentrations. Importantly, recent results have provided evidence that the Lp(a)-associated Lp-PLA_2 _may play an important role by degrading oxidized phospholipids that are preferentially sequestered on Lp(a) [35]. It is unlikely, however, that the Lp(a)-associated Lp-PLA_2 _activity might have influenced the plasma levels of this enzyme in ERA patients since the mean serum levels of Lp(a) in our patients as well as in controls are between 8.6 and 11.2 mg/dl – according to our previously published results, the plasma levels as well as the distribution of Lp-PLA_2 _between LDL and HDL can be influenced by the presence of Lp(a) only when plasma levels of this lipoprotein exceed 30 mg/dl [33].

An important observation of the present study is that ERA patients exhibited higher autoantibody titers against all types of mildly oxLDL (oxLDL_L_, oxLDL_P _and oxLDL_D_) at baseline compared with controls. One year of therapy with DMARDs resulted in a significant decrease of autoantibody titers against all types of oxLDL compared with the respective baseline values, a finding that could be attributed, at least partially, to the repression of the immune system activation due to immunointervention. Importantly, the antibody titers against oxLDL_D _are independently associated with ERA, thus providing evidence that such types of mildly oxLDL may be implicated in the pathophysiology of ERA. Indeed, previously published results showed that modified LDL with characteristics of minimally modified LDL, but not extensively oxidized LDL, is present in the synovial fluid of patients with rheumatoid arthritis [36].

Another important finding of the present study is that ERA patients exhibit low plasma HDL-cholesterol levels at baseline. According to our previously published results, this phenomenon could be at least partially attributed to the increased activity of the cholesterol ester transferring protein observed in plasma of ERA patients [23]. The present study further shows that HDL-cholesterol levels are inversely correlated with autoantibody titers against oxLDL_P _and oxLDL_D_. Furthermore, autoantibody titers against oxLDL_D _at baseline are inversely correlated with HDL-Lp-PLA_2_. Several studies over the past years have demonstrated that HDL exerts potent anti-inflammatory, antioxidant and antiatherogenic effects through its constituents. Among these constituents, the enzyme Lp-PLA_2 _may have a prominent role by degrading proinflammatory oxidized phospholipids formed on LDL during oxidation, thus limiting their accumulation on oxLDL [13]. The negative correlation between HDL-Lp-PLA_2 _activity and antibodies against oxLDL_D _found in the present study could therefore be attributed to the fact that oxLDL_D _compared with the other types of oxLDL is enriched in oxidized phospholipids that significantly contribute to the antigenicity of this type of oxLDL [37]. These phospholipids are substrates for HDL-Lp-PLA_2_; consequently the HDL-Lp-PLA_2 _activity could significantly lower the levels of oxidized phospholipids formed on oxLDL_D_, thus diminishing the antigenicity of this type of oxLDL. In addition to the HDL-Lp-PLA_2_, the Apo A-I content of HDL can bind oxidized lipids and remove them from LDL, therefore significantly contributing to the HDL-mediated retardation of LDL oxidation and thus the prevention of oxLDL proinflammatory activities [38].

According to our results, the low baseline levels of HDL-cholesterol and HDL-Lp-PLA_2 _activity in ERA patients are significantly increased after immunointervention, a phenomenon that could be at least partially attributed to the immunointervention-induced reduction in cholesterol ester transferring protein activity [23]. The elevation of HDL-cholesterol and HDL-Lp-PLA_2 _activity in ERA patients after immunointervention is associated with a reduction in the autoantibody titers against oxLDL. We may consequently suggest that the immunointervention-induced reduction in the autoantibody titers against oxLDL could be attributed not only to the repression of the immune system activation, but also to the increase in plasma HDL-cholesterol and HDL-Lp-PLA_2 _levels. Furthermore, this action of DMARDs may represent a potentially antiatherogenic effect of these drugs.

## Conclusion

The present study shows for the first time that ERA patients exhibit low plasma Lp-PLA_2 _and HDL- Lp-PLA_2 _activities and elevated autoantibody titers against mildly oxLDL. The low plasma Lp-PLA_2 _activity and the increased titers against oxLDL_D _are independently associated with ERA, suggesting an important role of these parameters in the pathophysiology of ERA. This hypothesis needs to be further supported by large-scale clinical studies.

## Abbreviations

Apo A-I = apolipoprotein A-I; Apo B = apolipoprotein B-100; CRP = C-reactive protein; DMARDs = disease-modifying antirheumatic drugs; ELISA = enzyme-linked immunosorbent assay; ERA = early rheumatoid arthritis; HDL = high-density lipoprotein; Lp(a) = lipoprotein (a); Lp-PLA_2 _= lipoprotein-associated phospholipase A_2_; HDL-Lp-PLA_2 _= high-density lipoprotein-associated phospholipase A_2_; IL = interleukin; LDL = low-density lipoprotein; oxLDL = oxidized low-density lipoprotein; oxLDL_D _= oxidized low-density lipoprotein in the decomposition phase; oxLDL_L _= oxidized low-density lipoprotein in the lag phase; oxLDL_P _= oxidized low-density lipoprotein in the propagation phase.

## Competing interests

The authors declare that they have no competing interests.

## Authors' contributions

ESL wrote the paper and performed the biochemical measurements. ANG participated in the selection of the patients and therapy. ECP contributed to the biochemical measurements and to writing the paper. AIP participated in the statistical analysis and in writing the paper. AAD participated in the selection of the patients and therapy. ADT conceived the idea for the study, participated in its design and coordination, and helped to draft the manuscript. All authors read and approved the final manuscript.

## Figures and Tables

**Table 1:** Clinical characteristics and lipid profile of early rheumatoid arthritis patients and controls

	Controls (*n *= 63)	Early rheumatoid arthritis patients	
		
		Baseline (*n *= 58)	Post-treatment (*n *= 56)
Sex (males/females)	20/43	14/44	13/43
Age (years)	58.4 ± 17.7	53.6 ± 15.3	54.7 ± 14.8
Body mass index (kg/m^2^)	25.8 ± 6.5	25.5 ± 3.3	25.8 ± 3.1
IgM rheumatoid factor (+/-)	0/0	45/13	44/13
C-reactive protein (mg/dl)	3 (1–5)	22 (7–104)	3 (1–5)^‡^
Disease activity score for 28 joint indices	-	5.8 ± 0.9	2.7 ± 1.0^‡^
Erythrocyte sedimentation rate (mm/h)	5.2 ± 3.1	48.0 ± 19.7	14.6 ± 8.7^‡^
Total cholesterol (mg/dl)	190.4 ± 33.9	216.5 ± 50.3*	228.1 ± 42.1**
Low-density lipoprotein-cholesterol (mg/dl)	126.5 ± 31.3	141.6 ± 42.3*	140.4 ± 32.4
High-density lipoprotein-cholesterol (mg/dl)	51.1 ± 7.4	47.5 ± 11.8^†^	60.7 ± 13.4^‡^
Triglycerides (mg/dl)	97.1 ± 28.3	133.0 ± 58.2*	131.5 ± 56.1
Apolipoprotein B-100 (mg/dl)	93 ± 19	103 ± 28*	105 ± 30
Apolipoprotein A-I (mg/dl)	144 ± 23	127 ± 27^†^	152 ± 22^‡^
Lipoprotein (a) (mg/dl)	10.6 (0.8–28.0)	11.2 (2.5–39.7)	8.6 (2.5–38.1)
Plasma lipoprotein phospholipase A_2 _activity (nmol/ml/min)	58.6 ± 14.0	47.1 ± 19.3^†^	45.7 ± 14.6
High-density lipoprotein-associated lipoprotein phospholipase A_2 _activity (nmol/ml/min)	3.4 ± 1.3	2.7 ± 1.0*	3.6 ± 1.3^‡^

Data presented as the mean ± standard deviation or as the median (range). *P < 0.001 and ^†^*P *< 0.0001 compared with controls, ^‡^*P *< 0.001 and **P < 0.01 compared with baseline values.

**Table 2:** Autoantibody titers against various types of oxidatively modified low-density lipoprotein (oxLDL) in early rheumatoid arthritis patients and controls

Antigen	Controls (*n *= 63)	Early rheumatoid arthritis patients	
		
		Baseline (*n *= 58)	Post-treatment (*n *= 56)
oxLDL in the lag phase	0.919 ± 0.271	1.131 ± 0.229*	0.872 ± 0.198^†^
oxLDL in the propagation phase	0.962 ± 0.289	1.223 ± 0.278*	1.045 ± 0.300^‡^
oxLDL in the decomposition phase	0.985 ± 0.376	1.375 ± 0.327*	1.144 ± 0.247**

Data presented as the mean ± standard deviation. *P < 0.0001 compared with controls, ^†^*P *< 0.001, ^‡^*P *< 0.01 and **P < 0.05 compared with baseline values.

**Table 3:** Univariate logistic regression analysis of factors associated with the presence of early rheumatoid arthritis

	Odds ratio	95% confidence interval	*P *value
Age	1.009	0.982–1.036	Not significant
Female gender	1.014	0.965–1.123	Not significant
Total-cholesterol	0.998	0.990–1.006	Not significant
Low-density lipoprotein-cholesterol	0.960	0.991–1.010	Not significant
High-density lipoprotein-cholesterol	0.969	0.931–1.008	Not significant
Triglycerides	0.998	0.991–1.006	Not significant
Apolipoprotein B-100	1.012	0.934–1.134	Not significant
Apolipoprotein A-I	0.985	0.876–1.078	Not significant
Antibodies against oxidized low-density lipoprotein in the lag phase	0.027	0.004–0.177	0.000
Antibodies against oxidized low-density lipoprotein in the propagation phase	0.029	0.005–0.171	0.000
Antibodies against oxidized low-density lipoprotein in the decomposition phase	0.034	0.008–0.152	0.000
Plasma lipoprotein phospholipase A_2 _activity	1.038	1.012–1.066	0.004
High-density lipoprotein-associated lipoprotein phospholipase A_2 _activity	1.021	0.897–1.245	Not significant

**Table 4:** Multivariate logistic regression analysis of factors associated with the presence of early rheumatoid arthritis

	Odds ratio	95% confidence interval (β)	*P *value
Antibodies against oxidized low-density lipoprotein in the lag phase	0.431	0.023–7.945	Not significant
Antibodies against oxidized low-density lipoprotein in the propagation phase	0.155	0.010–2.295	Not significant
Antibodies against oxidized low-density lipoprotein in the decomposition phase	0.047	0.008–0.282	0.001
Plasma lipoprotein phospholipase A_2 _activity	1.044	1.013–1.077	0.006

The model includes antibodies against oxidized low-density lipoprotein in the lag phase, the propagation phase and the decomposition phase, and plasma lipoprotein phospholipase A_2 _activity as defined from univariate analysis after adjustment for age and female gender.
